# A Primary Cell Culture‐Based Approach for Studying the Pathophysiology of Progressive Supranuclear Palsy (PSP)

**DOI:** 10.1002/alz70861_108801

**Published:** 2025-12-23

**Authors:** Priyanka Singh

**Affiliations:** ^1^ University of toronto, Toronto, ON Canada

## Abstract

**Background:**

PSP is characterized by **4‐repeat tau** accumulation in **neuron**al **neurofibrillary tangles, oligodendroglial coiled bodies and** in tufted **astrocytes** (a pathognomonic feature). In PSP, misfolded tau seeds the aggregation of tau throughout the brain in a prion‐like manner, with each cytopathology having a distinct, and sometimes overlapping pattern of distribution. The contribution of each of these three distinct cytopathologies to the accumulation and propagation of pathogenic tau throughout the PSP brain is not fully understood. To study this, we will develop a primary culture model of tau cytopathologies in PSP.

**Method:**

**Progenitor cells** were isolated from **P2‐P4 6hTau mice**, that express all six human tau isoforms. Cells were cultured in progenitor media and differentiated into **astrocytes** and **neurons** using media specific for each cell type and treated with human PSP and Alzheimer’s’ tau.

**Result:**

Astrocytes and neurons were labeled with GFAP and MAP2, respectively, to confirm their identity and purity. We will next introduce **human brain‐derived PSP tau** seeds into our cultures to investigate tau uptake and propagation. Initial studies will identify the optimal tau dose for aggregation and potency of tau in these cells. Then, using an **astrocyte‐neuron co‐culture model** we will assess how **each cell type** contributes to tau spread. Finally, we will explore the effects of tau accumulation on neurodegeneration.

**Conclusion:**

This study presents work towards developing an innovative cell culture model to investigate the role of different cytopathologies in the pathogenesis of PSP. The use of cell cultures enables the future incorporation of live imaging of tau trafficking between astrocytes and neurons. This model aims to deepen our understanding of PSP pathogenesis and provide a valuable platform for testing potential therapeutic strategies.

